# Conjunctival sac bacterial culture of patients using levofloxacin eye drops before cataract surgery: a real-world, retrospective study

**DOI:** 10.1186/s12886-022-02544-2

**Published:** 2022-07-30

**Authors:** Zhenyu Wang, Pei Zhang, Chen Huang, Yining Guo, Xuhe Dong, Xuemin Li

**Affiliations:** 1 Department of Ophthalmology, Peking University Third Hospital, Beijing, China; 2 Beijing Key Laboratory of Restoration of Damaged Ocular Nerve, Peking University Third Hospital, Beijing, China; 3 Medical Research Center, Peking University Third Hospital, Beijing, China

**Keywords:** Real world study, Antibiotic prophylactic therapy, Clinical factor, Levofloxacin

## Abstract

**Background:**

The use of antibiotics preoperatively is effective to decrease the incidence of ocular bacterial infections but may lead to high resistance rate, especially on patients with multi-risk clinical factors. This study systematically analyzed real-world data (RWD) of patients to reveal the association between clinical factors and conjunctival sac bacterial load and offer prophylaxis suggestions.

**Methods:**

We retrieved RWD of patients using levofloxacin eye drops (5 mL: 24.4 mg, 4 times a day for 3 days) preoperatively. Retrieved data included information on the conjunctival sac bacterial culture, sex, presence of hypertension and diabetes mellitus (DM), and history of hospital-based surgeries. Data was analyzed using SPSS 24.0.

**Results:**

RWD of 15,415 cases (patients) were retrieved. Among these patients, 5,866 (38.1%) were males and 9,549 (61.9%) females. 5,960 (38.7%) patients had a history of hypertension, and 3,493 (22.7%) patients had a history of DM. 7,555 (49.0%) patients had a history of hospital-based operations. There were 274 (1.8%) positive bacterial cultures. Male patients with hypertension and DM may be at increased risk of having positive bacterial cultures (*P* < 0.05). *Staphylococcus epidermidis* (*n* = 56, 20.4%), *Kocuria rosea* (*n* = 37, 13.5%), and *Micrococcus luteus* (*n* = 32, 11.7%) were the top 3 isolated strains. Most bacterial strains were resistant to various antibiotics except rifampin, and 82.5% (33 of 40 isolates) of *Staphylococcus epidermidis* isolates had multidrug antibiotic resistance. Numbers of culture-positive *Staphylococcus epidermidis* isolates in the male group and non-DM group were greater than those in the female and DM groups, respectively. *Micrococcus luteus* (*n* = 11, 8.8%) was found less frequently in non-hypertension group than in hypertension group.

**Conclusion:**

Sex (Male) and the presence of hypertension and DM are risk factors for greater conjunctival sac bacterial loads. We offer a prophylactic suggestion based on the combined use of levofloxacin and rifampin. However, this approach may aggravate risk of multidrug resistance.

## Background

Commensal bacterial flora in the conjunctival sac is a potential risk factor for infection after intraocular surgeries [1, 2]. A broad range of commensal bacteria have been identified at the ocular surface of patients with infectious eye disease and have been reported to contribute to the occurrence of endophthalmitis after cataract surgery [2–4]. According to the study of Durand [5], Coagulase-negative *Staphylococci* (70% of cases), *Staphylococcus aureus* (10%), and *Streptococcus spp.* (9%) are the major pathogens responsible for endophthalmitis cases after cataract surgery. Without effective preoperative examination and prevention, the bacteria mentioned above may lead to endophthalmitis, a devastating eye infection which can cause irreversible blindness in the infected eye within hours or days of symptom onset [5].

The use of antibiotics is an effective strategy to significantly decrease the incidence of ocular bacterial infections (positive swabs). Among all kinds of antibiotics, levofloxacin (which belongs to quinolones and fluoroquinolones) has been proved to have well-established efficacy and tolerability in the treatment of external ocular infections caused by both Gram-positive and Gram-negative bacteria [6–13]. However, with the widespread use of antibiotics, the resistance rate of bacteria towards antibiotics (including levofloxacin) has gradually increased, which has become a severe threat to public health [14–18]. It becomes even worse with a concomitant decline in the development of novel antibiotics and the emergence of multidrug-resistant strains [19, 20]. Moreover, patient-related risk factors such as older age, sex (male), the presence of hypertension and/or diabetes mellitus (DM), and a history of hospital-based surgery may be associated with increasing bacterial load and the emergence of multidrug-resistant bacteria [1]. However, the species and characteristics of multidrug-resistant bacteria in human conjunctival sac have not been systematically summarized.

According to The Food and Drug Administration (FDA), real-world data (RWD) is defined as all data relating to patient health status and/or the delivery of health care, routinely collected from a variety of sources. Moreover, real-world evidence (RWE) is the clinical evidence regarding the usage and potential benefits or risks of a medical product, derived from the analysis of RWD [21]. By studying RWE, clinicians can optimize currently available therapies or develop new prophylactic strategies [22]. It provides support for us to further study the characteristics of levofloxacin resistant bacteria in conjunctival sac.

In the current study, we searched the related literature and reviewed the results of conjunctival sac bacterial cultures of patients that had used Cravit (levofloxacin eye drops, Santen Pharmaceutical Co., Ltd) for antibiotic prophylactic therapy before cataract surgery. With the exception of data from the literature, all RWD were collected in Peking University Third Hospital from 2016 to 2019. By calculating the positive rate, analyzing positive strains and their drug sensitivity, as well as classifying results by clinical factors that may affect the positive rate of cultures, we revealed the association between different clinical factors and the conjunctival sac bacterial load. Further, by analyzing the results we confirmed the necessity for antibiotic use before cataract surgeries and offered prophylaxis suggestions and references.

## Methods

### Ethical approval and consent to participate

All participants provided written informed consent, consistent with the tenets of the Declaration of Helsinki. Peking University Third Hospital Medical Ethics Committee approved all procedures carried out in this study, including the procedure of accessing the clinical/personal patient data used in our research (approval number: M2019432).

### Data screening and selection

We included all medical records and related literature data and obtained RWD including basic patient information and conjunctival sac bacterial culture information of patients that had used Cravit (levofloxacin eye drops 5 mL: 24.4 mg, Santen Pharmaceutical Co., Ltd) for antibiotic prophylactic therapy before cataract surgeries. Literature on prophylactic therapy using other antibiotics or povidone-iodine (PVI) was also reviewed and summarized for comparison. For medical records, we restricted the inclusion criteria to patients with cataracts that had visited Peking University Third Hospital from 2016 to 2019. For published literature, the keywords used were “antibiotics”, “prophylactic therapy”, and “cataract surgery”. We restricted the inclusion criteria to observational cohort studies only. The timing of publication was restricted to the last 10 years (2009–2019). Any study published prior to the last 10 years was considered as outdated and was excluded. Moreover, studies that lacked information regarding age, sex, and previous medical history of patients and were not focused on the conjunctival sac bacterial culture of patients undergoing antibiotic prophylactic therapy were excluded. Publications were also excluded if the concentration of levofloxacin used was different from that in the current study. All relevant literature not included were summarized and compared with our study on the clinical effects of antibiotics and bacterial resistance to them.

### Data extraction

After screening medical records and publications, we extracted detailed data including the preoperative conjunctival sac bacterial culture of patients using Cravit, patient sex, presence of hypertension and/or DM, and history of hospital-based surgeries. All conjunctival sac bacterial culture samples were only collected and isolated from patients who had come for cataract surgery and had used Cravit preoperatively, 4 times a day for 3 days, from 2016 to 2019. For patients who underwent bilateral operations, we only conducted the cataract surgery on one eye at a time. The interval between the left eye operation and right eye operation of each patient was more than one month. The medical records of the first-eye surgeries were retrieved. Patients were asked to only use topical antibiotics on the eyes that were to be operated. The isolates were all collected from the conjunctival sac of patients just before the operation and were identified using the Vitek-2 automated systems (bioMerieux, France). Antimicrobial susceptibility testing (AST) for tobramycin, ceftriaxone, erythromycin, vancomycin, leveofloxacin, ofloxacin, and rifampin was performed using the Kirby-Bauer (K-B) disk diffusion method according to the Clinical and Laboratory Standards Institute (CLSI) guideline.

### Data analysis

Data of patient basic information, results of conjunctival sac bacterial culture, and antimicrobial susceptibility testing were collected and recorded using Excel (Microsoft Office 2019; Microsoft Corporation, Redmond, WA, USA). All statistical analyses were conducted using SPSS 24.0 (International Business Machines Corp.). Considering the data frame, distribution and sample sizes of our results, multiple statistical approaches were applied in our studies. Comparison of the incidence of each clinical factor between culture-positive groups and culture-negative groups was performed using the chi-square test. Binary logistic regression analysis was also used to explore the association between clinical factors and the positive culture of conjunctival sac bacteria. The Kruskal–Wallis H test was conducted to analyze the results of K-B test. Due to the small sample sizes for some strains, the Kruskal–Wallis H test was only conducted on strains of 6 or more isolated samples with K-B test results. Culture-positive patients were divided into two groups according to the clinical factors that were associated with culture results. The presence of various bacteria and their AST results were compared using the chi-square test and Mann–Whitney U test. It was notable that since we involved multiple factors without clear pre-defined hypothesis, multiple testing correction were considered into the final *P* value threshold (*P* < 0.001). Statistical significance of other tests was defined as *P* < 0.05.

## Results

### Overall results

#### Patients and clinical factors

RWD of 15,415 cases, including conjunctival sac bacterial cultures, were retrieved. Because the concentration of levofloxacin used in the published literature was different from that in the medical records of our study, and there was a lack of information regarding age, sex, and previous medical history of patients, all RWD were retrieved from the medical records of patients from Peking University Third Hospital. Clinical factors that may affect conjunctival sac bacterial load of patients before cataract surgery are shown in Table 1. Among the total cases, there were 5,866 (38.1%) males and 9,549 (61.9%) females. There were 5,960 (38.7%) patients with a history of hypertension and 3,493 (22.7%) patients with a history of DM. The number of patients with a history of one or more hospital-based operations was 7,555 (49.0%). There were 169 (1.1%) patients who had undergone bilateral operations and only the medical records of the first-eye surgeries were retrieved.

**Table 1:** Clinical factors related to conjunctival sac bacterial load in patients before cataract surgery

	Conjunctival sac bacterial culture		χ^2^	*P*
	Positive (*n*^b^ = 274)	Negative (*n* = 15,141)		
Sex				
Male	137(50.0%)	5729(37.8%)	16.888	< 0.001*^a^
Female	137(50.0%)	9412(62.2%)		
Hypertension				
Yes	149(54.4%)	5811(38.4%)	29.054	< 0.001*
No	125(45.6%)	9330(61.6%)		
Diabetes mellitus				
Yes	88(32.1%)	3405(22.5%)	14.236	< 0.001*
No	186(67.9%)	11,736(77.5%)		
History of hospital-based surgeries				
Yes	131(47.8%)	7424(49.0%)	0.161	0.688
No	143(52.2%)	7717(51.0%)		

^a^*P < 0.05 in two-side χ^2^ test

^b^*n*: number of patients

There were 274 culture samples that were positive, suggesting that these patients had a greater conjunctival sac bacterial load. The positive rate was 1.8%. Among them, there were 37 samples that led to postoperative endophthalmitis eventually (0.2% of all samples). Male patients (*n* = 137, 2.3%) and patients with a history of hypertension (*n* = 149, 2.5%) or DM (*n* = 88, 2.5%) were at an increased risk of having positive bacterial cultures (*P* < 0.05), but the history of hospital-based surgeries may have had no influence (*P* > 0.05). Besides, the results of binary logistic regression analysis was shown in Table 2 and the logistic model was statistically significant (χ^2^(4) = 52.686, *P* < 0.001). Among the 4 independent variables included in the model, sex, presence of hypertension and DM were statistically significant (*P* < 0.05). The risk of positive culture of conjunctival sac bacteria in male was 1.677 times higher than that in female. The risk in patients with hypertension was 1.844 times higher than that in patients without hypertension. The risk in diabetic patients was 1.385 times higher than that in non-diabetic patients. There were only three patients who had undergone bilateral operations, and the interval between the left eye operation and right eye operation of each patient, as previously stated, was more than one month (Table 3).

**Table 2:** Binary logistic regression analysis of positive conjunctival sac bacterial culture in patients before cataract surgery based on clinical factors

	B^b^	SE^b^	Wald^b^	d*f*^b^	*P*	Odds Ratio	95%CI for Odds Ratio^b^	
							Lower	Upper
Sex^a^	0.517	0.122	17.849	1	< 0.001*^c^	1.677	1.319	2.131
Hypertension	0.612	0.127	23.378	1	< 0.001*	1.844	1.439	2.364
Diabetes mellitus	0.326	0.135	5.825	1	0.016*	1.385	1.063	1.805
History of hospital-based surgeries	-0.125	0.123	1.029	1	0.31	0.883	0.694	1.123
Constant	-4.55	0.124	1344.781	1	< 0.001*	0.011		

^a^Sex is for males compared to females

^b^*B* regression coefficient, *SE* standard error of the mean, *Wald* Wald test score, *df* degree of freedom, *95% CI of OR* 95% confidence interval of odds ratio

^c^*P < 0.05 in binary logistic regression analysis

**Table 3:** Summary of conjunctival sac bacteria of the patients who had undergone bilateral operations

Patient No	Sex	Age (y)	Left/Right Eye	Operation date	Isolated bacteria	Zoom diameter of K-B test of antimicrobial agent (mm)						
						Tobramycin	Ceftriaxone	Erythromycin	Vancomycin	Levofloxacin	Ofloxacin	Rifampin
1	Male	67	OD	2018/9/10	*Micrococcus luteus*	-	34	12	23	22	20	32
			OS	2018/10/11	Unidentifiable bacteria	16	28	10	30	30	24	34
2	Female	75	OD	2017/6/7	*Acinetobacter lwoffii*	10	10	7	8	14	12	15
			OS	2018/5/24	Unidentifiable bacteria	10	24	-	14	9	9	30
3	Female	72	OD	2017/12/7	Inactive biochemical spectrum	-	16	16	20	7	7	28
			OS	2018/1/14	*Kocuria kristinae*	-	28	17	22	-	-	28

#### Culture identification

**Fig. 1:**
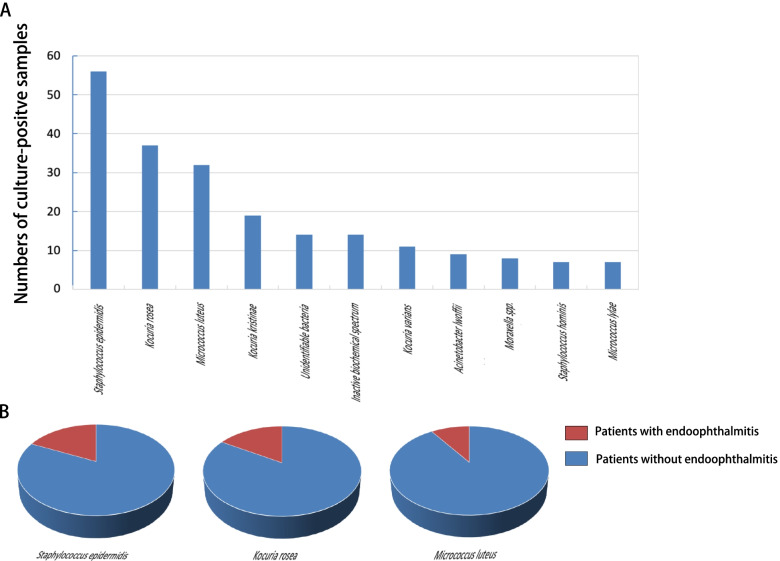
**A** Number of culture-positive samples for the top 10 identified bacterial strains. **B** Pie graphs showing the percentage of postoperative endophthalmitis for the three most common isolates

The top 10 species of culture-positive samples and the number of culture-positive samples of each species were shown in the Fig. 1A. Of all the 274 positive culture samples, *Staphylococcus epidermidis* (*n* = 56, 20.4%), *Kocuria rosea* (*n* = 37, 13.5%), and *Micrococcus luteus* (*n* = 32, 11.7%) were the three most frequently isolated strains, accounting for 45.6% of culture-confirmed cases. Furthermore, there were 19 positive samples in total that led to postoperative endophthalmitis for the three most common isolates (10 *Staphylococcus epidermidis* samples, 6 *Kocuria rosea* samples and 3 *Micrococcus luteus* samples). The percentage of postoperative endophthalmitis for the three most common isolates were shown as pie graphs in Fig. 1B.

#### Antimicrobial susceptibility testing

Among the 274 culture-positive samples, information on antimicrobial susceptibility testing using the K-B test was recorded for 234 (85.4%) samples and is summarized in Table 4. For *Staphylococcus epidermidis*, *Kocuria rosea*, *Kocuria kristinae*, *Kocuria varians*, *Micrococcus luteus*, *Micrococcus lylae*, *Moraxella spp.*, *Brevundimonas diminuta*, inactive biochemical spectra, and unidentifiable bacterial groups, there were statistically significant differences in resistance to different antimicrobial agents (*P* < 0.05). The zone diameters of rifampin in the K-B test were the largest, which means all these bacteria were most sensitive to rifampin.

**Table 4:** Number of isolated bacteria and K-B test results

Isolated bacteria	*n* (%)^a^	Number of K-B^b^ test results	Zone Diameter of Antimicrobial agent (mm)							*P*
			Tobramycin	Ceftriaxone	Erythromycin	Vancomycin	Levofloxacin	Ofloxacin	Rifampin	
***Staphylococcus spp.***	76(27.7%)									
*Staphylococcus epidermidis*	56(20.4%)	40	7(0)	22.5(19.25)	0(0)	15(14)	10.5(0)	7(0)	30(28)	< 0.001*^c^
*Staphylococcus hominis*	7(2.6%)	2	7(0)	23.5(23)	8.5(7)	19.5(19)	21(12)	20.5(12)	35(34)	-
*Staphylococcus warneri*	4(1.5%)	3	0(0)	20(18)	24(7)	14(12)	18(15)	18(11)	24(20)	-
*Staphylococcus capitalis*	2(0.7%)	1	13	18	0	17	10	0	30	-
*Staphylococcus aureus*	1(0.4%)	0								
*Staphylococcus auricularis*	1(0.4%)	1	20	22	26	15	22	20	30	-
*Staphylococcus caprae*	1(0.4%)	1	20	26	30	12	22	22	28	-
*Staphylococcus gallinarum*	1(0.4%)	0								
*Staphylococcus haemolyticus*	1(0.4%)	1	12	18	0	15	0	0	28	-
*Staphylococcus lentus*	1(0.4%)	1	18	32	20	16	12	12	30	-
*Staphylococcus saprophyticus*	1(0.4%)	1	20	22	22	14	20	16	24	-
***Kocuria spp.***	67(24.5%)									
*Kocuria rosea*	37(13.5%)	35	20(11)	28(24)	14(8)	21(20)	0(0)	0(0)	30(28)	< 0.001*
*Kocuria kristinae*	19(6.9%)	18	11(0)	28(23.5)	15(0)	24(18)	20(0)	16.5(0)	30(24.75)	< 0.001*
*Kocuria varians*	11(4.0%)	10	21(17.5)	22(17.5)	15.5(0)	24(20)	15(10.25)	18(11.5)	33.5(29.5)	< 0.001*
***Micrococcus spp.***	39(14.2%)									
*Micrococcus luteus*	32(11.7%)	29	11(8)	30(27)	12(8)	20(18)	20(16)	18(14)	30(29)	< 0.001*
*Micrococcus lylae*	7(2.6%)	7	13(10)	26(20)	10(8)	16(14)	18(14)	14(14)	30(28)	< 0.001*
***Acinetobacter spp.***	12(4.4%)									
*Acinetobacter lwoffii*	9(3.3%)	5	20(14)	14(5)	8(3.5)	9(8.5)	14(7)	13(11.5)	20(15.5)	-
*Acinetobacter baumannii*	1(0.4%)	0								
*Acinetobacter junii*	1(0.4%)	1	11	18	16	10	16	15	11	-
*Acinetobacter ursinensis*	1(0.4%)	1	24	24	24	8	22	24	20	-
***Streptococcus spp.***	9(3.3%)									
*Streptococcus granulosus*	4(1.5%)	4	8.5(0)	26(15)	18(14.5)	19(11.25)	18(15.25)	8(0)	28(17.5)	-
*Streptococcus sanguis*	3(1.1%)	3	7(0)	33(30)	30(15)	20(18)	20(19)	17(15)	26(24)	-
*Streptococcus mitis*	1(0.4%)	1	7	24	0	12	14	12	24	-
*streptococcus parasanguis*	1(0.4%)	1	10	24	0	14	9	9	30	-
***Moraxella spp.***	8(2.9%)	8	23(18.5)	31(26.5)	11(8.25)	14(12.25)	15(10.25)	12.5(0)	29(21.25)	0.003*
***Brevundimonas spp.***	7(2.6%)									
*Brevundimonas diminuta*	6(2.2%)	6	6(0)	25(19)	20(11.75)	13.5(11.75)	19(12.25)	14.5(7.5)	30(22.25)	0.024*
*Brevundimonas vesicularis*	1(0.4%)	1	0	24	22	16	24	22	28	-
***Neisseria spp.***	3(1.1%)									
*Neisseria sicca*	2(0.7%)	2	15.5(15)	22(20)	12.5(11)	0(0)	12(7)	7.5(0)	15(15)	-
*Neisseria longissima*	1(0.4%)	1	22	0	8	21	22	22	30	-
***Others***	53(19.3%)									
Inactive biochemical spectrum	14(5.1%)	14	12(0)	30(16.75)	16(12)	20(9.5)	11(0)	8.5(0)	27(15.5)	0.001*
Unidentifiable bacteria	14(5.1%)	12	19(14.5)	29.5(24.5)	17.5(10.5)	19(9.5)	23(17.75)	16(11)	26.5(17)	0.008*
*Dermatococcus westermani*	4(1.5%)	4	5(0)	31(21)	18(10.25)	23.5(22)	18(4)	7(0)	35.5(16)	-
*Coccus rhizophilus*	3(1.1%)	3	8(0)	26(14)	17(10)	22(18)	32(18)	14(0)	30(28)	-
*Rosella carioides*	3(1.1%)	3	14(9)	26(22)	15(10)	20(20)	22(16)	14(0)	26(20)	-
*Aerococcus viridans*	2(0.7%)	2	12(0)	32.5(28)	17(0)	20(16)	24(16)	19.5(13)	26(18)	-
*Aeromonas Salmonella*	2(0.7%)	2	20(18)	23(22)	19(19)	12.5(9)	28.5(27)	25.5(24)	21.5(20)	-
*Sphingomonas paucimobilis*	2(0.7%)	2	8(0)	21(14)	5(0)	23.5(17)	19(8)	12(0)	29(24)	-
*Alicyclobacillus acidoterrestris*	1(0.4%)	1	16	24	26	20	20	0	30	-
*Alloiococcus otitidis*	1(0.4%)	1	11	20	8	18	10	10	20	-
*Escherichia coli*	1(0.4%)	1	0	19	0	0	0	0	0	-
*Enterococcus faecalis*	1(0.4%)	1	13	0	20	16	18	15	0	-
*Morganella morganii*	1(0.4%)	1	13	21	0	0	20	16	9	-
*Myroides spp.*	1(0.4%)	1	0	30	16	15	12	0	28	-
*Klebsiella pneumoniae*	1(0.4%)	0								
*Roseomonas gilardii*	1(0.4%)	1	24	18	14	0	18	16	12	-
*Stenotrophomonas maltophilia*	1(0.4%)	1	0	0	11	0	20	20	0	-

^a^n(%): number of patients (percentage in the culture-positive group)

^b^K-B: Kirby-Bauer

^c^*P < 0.05 in the Kruskal–Wallis H test

Of the 234 identified cases with K-B test results, *Staphylococcus epidermidis* (*n* = 40, 17.1%) was the predominant organism. The results were retrieved and summarized in Table 5. According to the CLSI guideline, among 40 *Staphylococcus epidermidis* isolates, 100.0% were sensitive to vancomycin, 95.0% (38 of 40 isolates) to rifampin, 47.5% (19 of 40 isolates) to ceftriaxone, 37.5% (15 of 40 isolates) to tobramycin, 22.5% (9 of 40 isolates) to erythromycin, and 12.5% (5 of 40 isolates) to levofloxacin and ofloxacin.

**Table 5:** Results of K-B test of *Staphylococcus spp*

Antimicrobial agent		*Staphylococcus epidermidis*	*Staphylococcushominis*	*Staphylococcus warneri*	*Staphylococcus capitalis*	*Staphylococcus aureus*	*Staphylococcus auricularis*	*Staphylococcus caprae*	*Staphylococcus gallinarum*	*Staphylococcus haemolyticus*	*Staphylococcus lentus*	*Staphylococcus saprophyticus*
	n^a^	56	7	4	2	1	1	1	1	1	1	1
	Number of K-B^b^ test results	46	3	3	2	1	1	1	0	1	1	1
Tobramycin	R^c^	25	1	3	0	0	0	0	0	1	0	0
	I^c^	0	1	0	1	0	0	0	0	0	0	0
	S^c^	15	0	0	0	0	1	1	0	0	1	1
Ceftriaxone	R	14	0	2	1	0	0	0	0	1	0	1
	I	7	1	0	0	0	1	0	0	0	0	0
	S	19	1	1	0	0	0	1	0	0	1	0
Erythromycin	R	29	2	1	1	0	0	0	0	1	0	0
	I	2	0	0	0	0	0	0	0	0	1	1
	S	9	0	2	0	0	1	1	0	0	0	0
Vancomycin	R	0	0	0	0	0	0	0	0	0	0	0
	I	0	0	0	0	0	0	0	0	0	0	0
	S	40	2	3	1	0	1	1	0	1	1	1
Levofloxacin	R	34	1	1	1	0	0	0	0	1	1	0
	I	1	0	1	0	0	0	0	0	0	0	0
	S	5	1	1	0	0	1	1	0	0	0	1
Ofloxacin	R	35	1	1	1	0	0	0	0	1	1	0
	I	0	0	0	0	0	0	0	0	0	0	1
	S	5	1	2	0	0	1	1	0	0	0	0
Rifampin	R	2	0	0	0	0	0	0	0	0	0	0
	I	0	0	0	0	0	0	0	0	0	0	0
	S	38	2	3	1	0	1	1	0	1	1	1

^a^n: number of patients

^b^K-B test: Kirby-Bauer disk diffusion test

^c^R: resistance I: intermediate S: susceptible

**Fig. 2:**
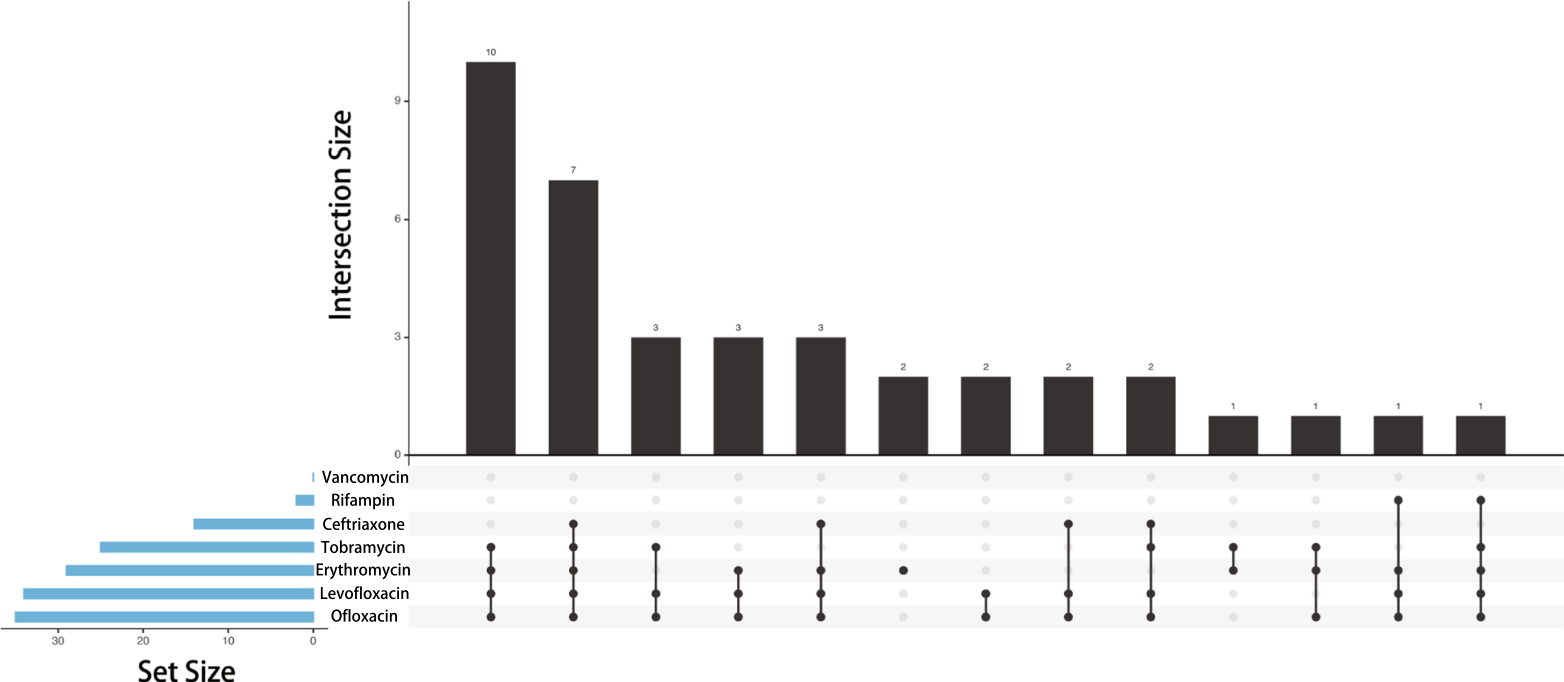
Upsetview of multidrug resistance of *Staphylococcus epidermidis*

It should be noted that the majority of *Staphylococcus epidermidis* (33 of 40 isolates, 82.5%) isolated samples had multidrug resistance to 3 kinds of antimicrobial agents or more. Further, 22.5% (9 of 40 isolates) were resistant to 3 kinds, 40.0% (16 of 40 isolates) to 4 kinds, and 20.0% (8 of 40 isolates) to 5 kinds. The Upsetview of multidrug resistance of *Staphylococcus epidermidis* is shown in Fig. 2.

### Subgroup classified by clinical factors

#### Sex

Among the 274 culture-positive samples, 50.0% (*n* = 137) were from male patients, and the rest (*n* = 137) were from females. For male culture-positive patients, *Staphylococcus epidermidis* (*n* = 31, 22.6%), *Kocuria rosea* (*n* = 18, 13.1%), *Kocuria kristinae* (*n* = 13, 9.5%), *Micrococcus luteus* (*n* = 11, 8.0%), and *Kocuria varians* (*n* = 7, 5.1%) were the 5 strains with the highest positive rates, accounting for 58.4% of culture-confirmed cases. For female culture-positive samples, *Staphylococcus epidermidis* (*n* = 25, 18.2%) was still the most prevalent culture-positive strain, followed by *Micrococcus luteus* (*n* = 21, 15.3%) and *Kocuria rosea* (*n* = 18, 13.1%). These 3 strains accounted for 46.7% of the culture-confirmed cases. It should be noted that the number of *Staphylococcus epidermidis*-positive isolates in the male patient group (*n* = 31, 22.6%) was more than that in the female patient group (*n* = 25, 18.2%), and there was significant difference between the two groups (χ2 = 7.139, *P* < 0.05). There was no significant difference in K-B results for various antimicrobial agents between the male and female patients.

#### Hypertension

Patients with hypertension had more positive culture results than those without hypertension (*P* < 0.05). Among culture-positive patients with hypertension, there were 18.1% (*n* = 27) with *Staphylococcus epidermidis*, 14.1% (*n* = 21) with *Micrococcus luteus*, and 12.1% (*n* = 18) with *Kocuria rosea*. For culture-positive patients without hypertension, *Staphylococcus epidermidis* (*n* = 29, 23.2%), *Kocuria rosea* (*n* = 19, 15.2%), and *Kocuria kristinae* (*n* = 11, 8.8%) were the 3 most prevalent strains. Compared to the hypertension patient group (*n* = 21, 14.1%), *Micrococcus luteus* (*n* = 11, 8.8%) was less frequently found in patients without hypertension. There was statistically significant difference between the two groups (χ2 = 9.829, *P* < 0.05).

As for K-B test results, the median zone diameter of *Staphylococcus epidermidis* for ofloxacin in the hypertension group (0 mm) was smaller than that in the non-hypertension group (9 mm), and there was a significant difference between two groups (*P* < 0.05). However, this could be related to use of levofloxacin preoperatively and requires careful analysis.

#### Diabetes mellitus

The 4 strains with the highest positive culture rates in the DM group were the same as the strains in the non-DM group, namely *Staphylococcus epidermidis* (*n* = 22, 25.0% of patients with DM; *n* = 34, 18.3% of patients without DM), *Kocuria rosea* (*n* = 12, 13.6% of patients with DM; *n* = 25, 13.4% of patients without DM), *Micrococcus luteus* (*n* = 12, 13.6% of patients with DM; *n* = 25, 13.4% of patients without DM), and *Kocuria kristinae* (*n* = 5, 5.7% of patients with DM; *n* = 14, 7.5% of patients without DM). The number of *Staphylococcus epidermidis*-positive samples of non-diabetic patients (*n* = 34, 18.3%) was greater than the number among diabetic patient samples (*n* = 22, 25.0%), and there was a significant difference between the two groups (χ2 = 8.865, *P* < 0.05). The information of K-B test results was also retrieved, and there was no significant difference between the diabetes group and non-diabetes group.

#### Comprehensive analysis of related clinical factors

**Fig. 3:**
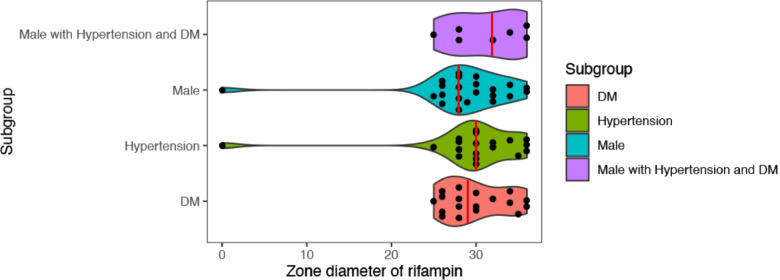
Violin plot of K-B test of *Staphylococcus epidermidis* against Rifampin. Each red line represents the median of each subgroup

After comprehensive analysis of all related clinical factors, we identified 27 (9.9% of all positive samples) male patients with both hypertension and diabetes mellitus. *Staphylococcus epidermidis* was the most detected strain (*n* = 9, 33.3%). The proportion of *Staphylococcus epidermidis* was highest in the male group (22.6%), the hypertension group (18.1%), and the DM group (25.0%). There were significant differences for various antimicrobial agents in the K-B test (*P* < 0.05), and the zone diameters of rifampin were largest of all the antimicrobial agents (median zone diameter was 32 mm). As shown in the Fig. 3, the median zone diameter of rifampin in samples from males with hypertension and DM (32 mm) was larger than that in the male group (28 mm), hypertension group (30 mm), and DM group (29 mm). There were no significant differences between groups (*P* > 0.05).

## Discussion

This study systematically retrieved RWD of 15,415 cases of patients that had used levofloxacin eye drops preoperatively. Data was retrieved from published literature from the last 10 years and from patients that had come to Peking University Third Hospital from 2016 to 2019. As we searched, there were several studies on conjunctival swab culture in cataract patients preoperatively without using antibiotic drops in the eyes (Table 6). According to the results, the positive rate of bacterial cultures of the conjunctival sac in cataract patients preoperatively without using antibiotic drops ranged from 48.3% to 74.0% [23–25]. Through 36 16S rRNA gene libraries from 45 samples of preoperative cataract patients, Deepthi et al. indicated that among all the 211 detected isolates in human conjunctival sacs, the most often detected genera were *Corynebacterium spp.* (*n* = 30, 14.93%), *Staphylococcus spp.* (*n* = 26, 12.94%), and *Cutibacterium spp.* (*n* = 23, 11.44%), followed by *Escherichia spp.* (*n* = 13, 6.47%) and *Acinetobacter spp.* (*n* = 12, 5.97%) [26]. In the current study, the results revealed that after topically applying levofloxacin preoperatively, the positive rate of bacterial cultures from the conjunctival sac were 1.8%, which was indicative of the strong antimicrobial effect of levofloxacin in application before cataract surgery. However, it should be noted that even if levofloxacin had been used four times a day for 3 days, the possibility of a positive conjunctival sac bacterial culture still remained. Due to residual bacteria in the conjunctival sac, culture-positive patients were still at risk of endophthalmitis and other infectious diseases. Historically, the incidence of post-cataract surgery endophthalmitis ranges from 0.03% to 0.70% which could lead to serious consequences [27, 28]. As shown in Table 7, there are several major pathogens isolated from conjunctival sac of patients with post-cataract surgery endophthalmitis [5, 18, 29–38]. Among them, Gram-positive bacteria is the major pathogen and Coagulase-negative *Staphylococci* is the most frequently isolated strain [5, 18, 32, 33, 36–38]. According to Egrilmez et al., Coagulase-negative *Staphylococci* shows resistance rates of more than 30% for fluoroquinolone and methicillin [39]. In addition to endophthalmitis, it can also lead to other infectious diseases including bacterial keratitis. Without effective antibiotic prophylactic therapy, patient may be at risk of potentially vision-threatening infection.

**Table 6:** Summary of studies on conjunctival swab culture in cataract patients preoperatively without using antibiotic drops in the eyes

Year of Publication	Patients/eye number	Positive rate of cultured samples	Major Pathogen	References
1999	49 patients/49 eyes	69.4%	*Staphylococcus epidermidis* and other coagulase-negative *staphylococcus* species	Tervo et al. [23]
2003	100 patients/100 eyes	74.0% (twenty six of the 100 cultures were “sterile”)	Coagulase-negative *staphylococcus*	Ferguson et al. [24]
2012	56 patients/112 eyes	48.3% (54 eyes)	Coagulase-negative *staphylococcus* (44 eyes, 81.5% of positive samples)	Keshav et al. [25]

**Table 7:** Summary of major pathogens involved in post-cataract surgery endophthalmitis

Year of Publication	Major Pathogen	References
2005	*Pseudomonas aeruginosa*	Kenchappa et al. [29]
2009	*Pseudomonas aeruginosa*	Pinna et al. [30]
2009	*Stenotrophomonas maltophilia*	Horster et al. [31]
2011	Gram-positive bacteria (65.2%)	Ding et al. [32]
2013	Coagulase-negative *Staphylococci*	Durand [33]
2015	Coagulase-negative *Staphylococci*	Chiquet et al. [18]
2015	*Stenotrophomonas maltophilia* (57.1%)	Ji et al. [34]
2015	*Pseudomonas aeruginosa*	Priya et al. [35]
2017	Coagulase-negative *Staphylococci* (70%), *Staphylococcus aureus* (10%), *Streptococci* (9%)	Durand [5]
2017	Gram-positive bacteria (96%, Coagulase-negative *Staphylococci* is the most, accounting for 52%)	Slean et al. [36]
2018	Gram-positive bacteria (95%), including Coagulase-negative *Micrococci* (*Staphylococcus*)70%, *Staphylococcus aureus* 10%, *Streptococcus* species 9%, *Enterococcus* species 2.2%	Rahmani et al. [37]
2018	Gram-negative bacteria (5%), including *Pseudomonas*, *Proteus*, and *Haemophilus Influenzae*	Rahmani et al. [37]
2019	Gram-positive bacteria (89%, *Staphylococcus* is the most, accounting for 67%)	Slipa-Archa et al. [38]

According to our results, *Staphylococcus epidermidis*, *Kocuria rosea*, and *Micrococcus luteus* were the 3 strains with the highest culture-positive rates after usage of levofloxacin eye drops for 3 days preoperatively. All of these bacteria belong to the *Micrococcaceae* family and are commensals, which can be found on human skin, mucous membranes, and the conjunctival sac [40, 41]. They can cause opportunistic infections, requiring considerable attention [42]. *Staphylococcus epidermidis* is considered non-pathogenic. However, patients with a compromised immune system are often at risk of being infected. Characteristically, infections caused by *Staphylococcus epidermidis* are often chronic, which contrasts the acute infections caused by *Staphylococcus aureus* [43]. The pathogenesis of *Staphylococcus epidermidis* infection usually involves the formation of biofilms and phenol-soluble modulins which can kill human red and white blood cells [44–46]. It has been reported that *Staphylococcus epidermidis* cause biofilm growth on intravenous catheters and medical prostheses [47]. Thus, patients with *Staphylococcus epidermidis* are at risk of infection after implantation of intraocular lenses during cataract surgery. Besides, *Kocuria rosea* and *Micrococcus luteus* can also cause infectious disease in immunocompromised hosts. It has been reported that *Kocuria rosea* can cause meningitis, canaliculitis, endocarditis, and descending necrotizing mediastinitis [48–54]. As an opportunistic pathogen, *Micrococcus luteus* can also cause serious infections, such as endocarditis and brain abscess [55, 56]. Our study shows that patients with certain clinical factors (male, the presence of hypertension or diabetes mellitus) are at risk of having a greater conjunctival sac bacterial load, which has been confirmed in previous studies [57–60]. These factors are often present in patients, which may lead to immunocompromised hosts and resulting ocular opportunistic infections caused by the above-mentioned bacteria[1]. It is therefore suggested that ophthalmologists pay more attention to patients with any of these three clinical factors. As for the antibiotic resistance of conjunctival sac bacteria, we found that the resistance of *Staphylococcus epidermidis* against ofloxacin in the hypertension group was stronger than in the non-hypertension group (*P* < 0.05). However, the result cannot explain a direct relationship between hypertension and antibiotic resistance of bacteria and how these relate to the preoperative use of levofloxacin. Levofloxacin, a fluoroquinolone, is an isomer of ofloxacin [61]. By using levofloxacin preoperatively, ofloxacin-sensitive bacteria were widely eliminated in patients, and the ratio of ofloxacin-resistant bacteria in patient conjunctival sacs was relatively increased. This may have influenced the results of the current study.

The fact that there still were culture-positive samples after three days of antibiotic prophylactic treatment with levofloxacin shows that, in addition to a high conjunctival sac bacterial load, another possible reason could be the drug resistance of these bacteria. With the widespread use of antibiotics, antimicrobial resistance rates have gradually increased [19, 20]. In the current study, several kinds of bacterial strains were reported as resistant to antimicrobial agents, especially to levofloxacin and ofloxacin. Among them, several *Staphylococcus epidermidis* isolates had multidrug resistance to antimicrobial agents. It is commonly believed that antimicrobial resistance is higher in *Staphylococcus epidermidis* than in other Coagulase-negative *Staphylococcus spp.* [62]. The resistance of these bacterial strains against levofloxacin has been confirmed in several studies and has raised questions regarding the use of particular antimicrobial agents for routine prophylaxis [14–18].

In order to further decrease the conjunctival sac bacterial load through antibiotic prophylactic therapy, we need to carefully consider combinations of other effective antimicrobial agents. Our study suggests that rifampin would be a good choice for better topical prophylactic therapy, since most bacteria were sensitive to that agent. Rifampin belongs to rifamycins and has activity against several types of bacteria. Rubio et al. pointed out that 83.9% of conjunctival sac bacteria were sensitive to rifampin. Rifampin was the most effective for the eradication of the whole, predominantly Gram-positive, flora [63]. According to Chojnacki et al., the rifampin plus polymyxin B-trimethoprim combination demonstrated synergistic antimicrobial activity towards ocular clinical *Staphylococcus aureus* and *Pseudomonas aeruginosa* isolates, a low spontaneous resistance frequency, and in vitro bactericidal kinetics and antibiofilm activities equal to or exceeding those of moxifloxacin [64]. Compared to literature on the clinical effects of other antibiotics (Table 8), our study revealed a higher sensitivity of conjunctival sac bacteria towards rifampin[7, 65–75]. Further, there was not enough evidence for side effects of the topical application of rifampin at low concentrations.

**Table 8:** Summary of antibiotic studies

Antibiotic	Patients/eye number	Effects	References
Cefuroxime	2,434,008 patients/ 3,351,401 eyes	The intracameral injection of cefuroxime at the end of cataract surgery is associated with a lower risk of postoperative endophthalmitis and is safe for patients with or without a perioperative capsular rupture	Daien et al. [65]
Gatifloxacin	204,515 patients	Intracameral antibiotic was more effective for preventing post-cataract extraction endophthalmitis than topical antibiotic alone	Herrinton et al. [66]
Third-generation fluoroquinolone, fourth-generation fluoroquinolone, tobramycin	75,318 eyes	Preoperative and the operation day antibiotics have no influence on postoperative endophthalmitis rate	Rudnisky et al. [67]
Levofloxacin 1.5%	96 patients/96 eyes	The positive rate of conjunctival sac bacterial culture decreases from 78.1% to about 11.5%; Levofloxacin has good safety and effectiveness in conjunctival sac eradication, especially for Gram-positive bacteria, but is not as effective for Propionibacterium acnes	Suzuki et al. [7]
Ciprofloxacin 0.3%	46 patients/46 eyes	The administration of 0.3% ciprofloxacin significantly reduced colony-forming units compared with the control group (P < 0.05)	Carron et al. [68]
Levofloxacin, gentamycin, chloramphenicol, fusidic acid	464,996 eyes	Among patients treated with preoperative antibiotics and intracameral antibiotics, there were eight cases of postoperative endophthalmitis (0.017%), P = 0.29 vs. group with intracameral antibiotic alone. Addition of preoperative antibiotic does not reduce the risk of postoperative endophthalmitis	Friling et al. [69]
Antibiotic (ofloxacin) vs. antiseptic (hexamidine di-isetionate) eye drops	58 patients/60 eyes	Antibiotic and antiseptic eye drops had similar results in disinfection of the conjunctival sac	Vaninbroukx [70]
Moxifloxacin 0.5%	144 patients	The positive rate of conjunctival sac bacterial culture decreases to about 35%.1 day and 3 days of therapy with moxifloxacin had the same efficacy in decontamination of the conjunctival sac, but 1 day of prophylactic treatment with moxifloxacin resulted in a significant increase of resistance to fluoroquinolones; moxifloxacin treatment for 3 days did not cause an increase in resistance	He et al. [71]
Moxifloxacin 0.5%	148 patients	93% reduction of CFU; Application of moxifloxacin on the day of surgery is effective in reducing colony-forming units	Vasavada [72]
Gatifloxacin 0.3% vs. moxifloxacin 0.5%	220 patients	There was no difference between the two antibiotics	Bucci et al. [73]
Netilmicin	56 patients/56 eyes	Coagulase negative staphylococci was positive in 9.93% of patients after treatment; 83.9% of samples had no bacterial growth; Staphylococcus aureus was eliminated after treatment	Aslan et al. [74]
Gatifloxacin	60 patients/120 eyes	Reduction of CFU: 67 to 28% (1 day); 60 to 37% (1 h); 67 to 18% (1 day, 1 h)	Moss et al. [75]

Although rifampin is a good choice for combination therapy, it may lead to multidrug resistance and more severe consequences, including fever, headache, orange tears, skin redness or rash (allergic reaction) and other symptoms. Usage of multiple antimicrobial agents can effectively reduce bacterial load in the conjunctival sac. However, more resistant strains can also develop as a result of combined treatment. Therefore, simply adding more antimicrobial agents is an unsustainable strategy for improving antibiotic prophylactic therapy. Furthermore, patients may be at a greater risk of infectious diseases, and the proportion of antibiotic abuse may be higher due to clinical factors. The bacterial flora of the ocular surface may have already been multidrug-resistant in these patients. Thus, local application of multiple antibiotics may aggravate the risk of multidrug resistance.

Alternatively, we advocate a variety of other methods for decreasing the conjunctival sac bacterial load without using more antibiotics. Usage of povidone iodine (PVI) for irrigation during operation can reduce the bacterial burden in the conjunctival sac and has been proven as effective [76]. According to available literature (Table 9), the irrigation with high concentrations of PVI (5%-10%) can effectively decrease the conjunctival bacterial flora. PVI (5%) solution does not increase antimicrobial resistance and has no adverse effects. Low-concentration PVI (0.05%) irrigation of the conjunctival sac for 30 s can achieve a low bacterial contamination rate and reduce damage to the ocular surface. Levofloxacin can enhance the effectiveness of conjunctival sac irrigation with PVI solution [9, 77–88]. Compared to the preoperative use of topical antibiotics, the use of PVI can achieve the same degree of elimination of conjunctival sac bacteria. However, appropriate PVI concentration and irrigation duration should be precisely controlled, or it may cause damage to the ocular surface. Preoperative topical antibiotic treatment could be used as an additional method for further elimination of conjunctival sac bacteria.

**Table 9:** Summary of povidone-iodine studies (PVI)

Analyzed chemotherapeutic	Patients/eyes number	Effects	References
0.05% PVI	90 patients/90 eyes	0.05% PVI irrigation of the conjunctival sac for 30 s can achieve a low bacterial contamination rate. Importantly, it reduced the damage of the ocular surface, which is beneficial for the recovery of ocular surface function	Fan et al. [77]
0.3% PVI	51 participants	Preoperative treatment with long-term, low-concentration PVI applied via a depot device to the fornix inferior seems to be an easy and effective way to reduce the number of bacterial colonies in the conjunctiva (66.7% to 23.4%)	Wass et al. [78]
10% PVI 3 min	604 patients	Implementation of a preoperative prophylaxis protocol that used PVI 10% with a 3-min exposure time can be performed in clinical practice. The 3-min exposure time had no adverse sequelae	Nguyen et al. [79]
0.33% PVI	99 patients/ 198 eyes	Timely iodine irrigation can serve as a simple and useful adjunctive disinfection step in cataract surgery. The bacterial DNA copy number decreased from 1.7 ± 0.5 × 103 to 1.7 ± 0.6 × 104	Matsuura et al. [80]
5% PVI	13 eyes before serial intravitreal injection; 48 cultures performed	5% PVI solution does not increase antimicrobial resistance and has no adverse effects on the conjunctival bacterial flora	Hsu et al. [81]
Levofloxacin 0.3% + PVI 1 vs. 5 vs. 10%	271 patients	10% PVI solution was most effective in the reduction of the bacterial flora in the conjunctival sac. Most common isolated bacteria were coagulase-negative Staphylococcocus spp.	Li et al. [82]
10% PVI drops vs. irrigation of the conjunctival sac with 1% PVI	242 patients/263 eyes	Three drops of 10% PVI prior to surgery, followed by preoperative irrigation of the conjunctiva with 1% PVI, provides additional reduction in conjunctival sac bacterial cultures (positive rate reduced from 69–93% to 1–16%)	Nentwich et al. [83]
0.3% Ciprofloxacin vs. 0.3% Ofloxacin vs. 5% PVI	164 patients/ 164 eyes	Ciprofloxacin was the most effective in bacterial eradication (72.2% to 8.0%). PVI solution (75.4% to 22.7%) was more effective than Ofloxacin (59.6% to 33.4%)	Coskun et al. [84]
5% PVI	221 patients/ 224 eyes	5% PVI is effective for the reduction of bacterial flora and reduction of bacterial growth (from 73.2 to 12.5%)	Quiroga et al. [85]
5% PVI solution	54 patients/ 54 eyes	5% PVI for 3 min significantly reduced positive cultures; A reduction of the proportion of positive swabs from 87 to 30%	Carrim et al. [86]
Moxifloxacin 5 and 5% povidone- iodine (PVI) vs. 5% PVI	464 patients	Therapy with 5% PVI solution is effective in the reduction of positive conjunctival cultures. Adding 0.5% moxifloxacin had no significant effect in the reduction of conjunctival bacteria	Halachmi-Eyal et al. [87]
0.5% Levofloxacin (LVFL) and 1% PVI solution vs. 1% PVI solution alone	140 patients/ 140 eyes	Levofloxacin enhanced effectiveness of irrigation of the conjunctival sac with PVI solution	Min˜o de Kaspar et al. [88]
Levofloxacin 0.5% (LVFX) ophthalmic solution vs. 16-fold dilution of PVI solution vs. sixfold dilution of polyvinyl alcoholiodine (PAI) solution	272 patients/ 272 eyes	3-day therapy with LVFX and eyewash with diluted iodine solution is effective in disinfection of the conjunctival sac. It is impossible to eliminate all of bacteria from the conjunctival sac (Propionibacterium acnes and Staphylococcus epidermidis)	Inoue et al. [9]

We must admit that our study still has limitations. Due to a lack of information, some statistical analyses could not be conducted and we may not provide unexpected results. Not all clinical factors related to conjunctival sac bacterial load were analyzed in our study due to missing data, including age, history of cancer, and screening for infectious diseases. These factors cannot be ignored and would have to be investigated in a follow-up study. However, this limited result can still arouse our attention to the drug-resistance of conjunctival sac bacteria and provide suggestions for preventive treatment.

## Conclusions

Male and the presence of hypertension and diabetes mellitus are clinical risk factors for a greater conjunctival sac bacterial load. In order to decrease the conjunctival sac bacterial load for the prevention of possible infections, we offer a prophylaxis suggestion based on RWD, namely the combined use of levofloxacin and rifampin. However, such combined therapy but may aggravate the risk of multidrug resistance. Therefore, alternative ways should be suggested.

## Data Availability

The datasets used and analyzed during the current study are available from the corresponding author on reasonable request.

## Abbreviations

RWD: Real-world data;
DM: Diabetes mellitus;
FDA: The Food and Drug Administration;
RWE: Real-world evidence;
PVI: Povidone-iodine;
AST: Antimicrobial susceptibility testing;
K-B: Kirby-Bauer;
CLSI: Clinical and Laboratory Standards Institute
